# TNIK Inhibition Has Dual Synergistic Effects on Tumor and Associated Immune Cells

**DOI:** 10.1002/adbi.202200030

**Published:** 2022-06-08

**Authors:** Jaehee Kim, Juhyun Oh, Hannah M. Peterson, Jonathan C.T. Carlson, Mikael J. Pittet, Ralph Weissleder

**Affiliations:** Center for Systems Biology, Massachusetts General Hospital, 185 Cambridge St, CPZN 5206, Boston, MA 02114, USA; Center for Systems Biology, Massachusetts General Hospital, 185 Cambridge St, CPZN 5206, Boston, MA 02114, USA; Center for Systems Biology, Massachusetts General Hospital, 185 Cambridge St, CPZN 5206, Boston, MA 02114, USA; Center for Systems Biology, Massachusetts General Hospital, 185 Cambridge St, CPZN 5206, Boston, MA 02114, USA; MGH Cancer Center, Massachusetts General Hospital and Harvard Medical School, Boston, MA 02114, USA; Center for Systems Biology, Massachusetts General Hospital, 185 Cambridge St, CPZN 5206, Boston, MA 02114, USA; Department of Pathology and Immunology, University of Geneva, Agora Cancer Center, Rue du Bugnon 25A, 1000, Lausanne, Switzerland; Ludwig Institute for Cancer Research, Lausanne, Switzerland; Center for Systems Biology, Massachusetts General Hospital, 185 Cambridge St, CPZN 5206, Boston, MA 02114, USA; MGH Cancer Center, Massachusetts General Hospital and Harvard Medical School, Boston, MA 02114, USA; Department of Systems Biology, Harvard Medical School, 200 Longwood Ave, Boston, MA 02115, USA

**Keywords:** colorectal cancer, immunogenic cell death, immunotherapy, kinase inhibitors, TNIK

## Abstract

Treatment with checkpoint inhibitors can be extraordinarily effective in a fraction of patients, particularly those whose tumors are pre-infiltrated by T cells. In others, efficacy is considerably lower, which has led to interest in developing strategies for sensitization to immunotherapy. Using various colorectal cancer mouse models, it is shown that the use of Traf2 and Nck-interacting protein kinase inhibitors (TNIKi) unexpectedly increases tumor infiltration by PD-1^+^ CD8^+^ T cells, thus contributing to tumor control. This appears to happen by two independent mechanisms, by inducing immunogenic cell death and separately by directly activating CD8. The use of TNIKi achieves complete tumor control in 50% of mice when combined with checkpoint inhibitor targeting PD-1. These findings reveal immunogenic properties of TNIKi and indicate that the proportion of colorectal cancers responding to checkpoint therapy can be increased by combining it with immunogenic kinase inhibitors.

## Graphical Abstract

Kim et al. reports dual synergistic effects of TNIK inhibition on tumor and associated immune cells. TNIK inhibition increased tumor infiltration of PD-1^+^ CD8^+^ T cells, thus contributing to tumor control. The use of TNIK inhibitors achieved complete tumor control in 50% of mice when combined with immune checkpoint inhibitor targeting PD-1.

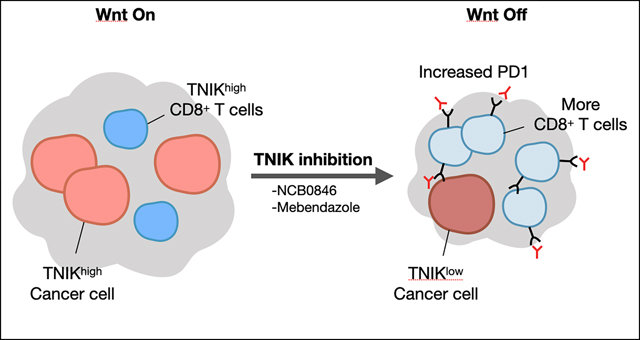

## 1. Introduction

Colorectal cancer (CRC) ranks as one of the most prevalent cancers worldwide, with 1.8 million new cases and 881 000 deaths in 2018 worldwide.^[1,2]^ Fortunately, the mortality rate has continuously declined^[2]^ due to advances in screening and therapy.^[3]^ Surgical intervention remains the treatment of choice for localized disease. In patients with advanced tumors, radiotherapy, chemotherapy, and targeted therapies are available.^[3]^ Despite these advances, there remains a need for more effective interventional approaches.^[4]^

Various signaling pathways can promote CRC initiation, progression, and/or dissemination. These pathways include Wnt/*β*-catenin, Notch, Hedgehog, Transforming growth factor *β* (TGF-*β*)/SMAD, Phosphoinositide 3-kinase(PI3K)/AKT, and RAS/RAF, all of which are potential therapeutic targets. In addition, Traf2 and Nck-interacting protein kinase (TNIK) was recently identified as a regulatory component of the *β*-catenin and T-cell factor-4 (TCF-4) transcriptional complex. Several small molecule compounds targeting this protein (NCB0846 (NCB), mebendazole (MBZ), and others have shown to have antitumor effects.^[5]^ In melanoma, it has been reported that tumor-intrinsic active *β*-catenin signaling results in T-cell exclusion and resistance to anti-PD-L1/anti-CTLA-4 monoclonal antibody therapy.^[7]^ It has also been reported that TNIK inhibitors (TNIKi) can enhance T-cell differentiation toward effector cells in acute infection.^[6]^ With the possible differential impacts on both tumor and immune cells, the in vivo effects of TNIKi are complex and there is no comprehensive understanding of how it affects the CRC microenvironmentt.

We recently developed a multiplexed immunophenotyping method (FAST) that enables serial sampling and in-depth analysis of cells in the tumor microenvironment by fine needle aspiration (FNA).^[8]^ Using this method, we have interrogated the immune landscape in murine CRC models and uncovered how it changes after exposure to different TNIKi compounds. Together with additional in vivo and in vitro studies, the results reveal that TNIKi treatment can trigger potent CD8^+^ T cell-mediated antitumor responses by inducing immunogenic tumor cell death,^[9]^ further promoting CD8^+^ T cell recruitment. Finally, we observed that TNIKi-induced activated CD8^+^ T cells expressed PD-1, providing a rationale for testing a TNIKi/anti-PD-1 combination regimen that yielded profound tumor control.

## 2. Results

### 2.1. Murine CRC Models Show Tumoral TNIK Protein Expression and Inadequate Immune Cell Infiltration

Wnt/*β*-catenin signaling is perturbed in over 90% of patients with CRC, prompting us to focus on immunocompetent cancer mouse models that also feature activation of this pathway. Prior work has shown that Wnt is activated in MC38^[10]^ and CT26,^[11]^ and that was indecently confirmed by our studies. Mice implanted with syngeneic tumor model MC38 or CT26 cells develop carcinomas with both pathophysiological and molecular features of the human disease.^[12–14]^ Evaluation of the MC38 tumor-bearing mice revealed the presence of TNIK in tumor cells (Figure S1, Supporting Information). Importantly, the tumors were largely devoid of CD8^+^ and CD4^+^ T cells.^[15]^ In addition, previous studies in the MC38 model indicated the presence of other cells with immunosuppressive functions, including tumor-associated macrophages.^[8,15,19]^

To test the efficacy of TNIK inhibition in these models, we focused on two prototypical inhibitors: NCB0846 (NCB), which has been tested preclinically^[5]^ and mebendazole (MBZ), which is approved by the FDA for treatment of helminth infections. Figure S2 (Supporting Information) summarizes the overall study design, in which the potency and impact of the two compounds were characterized in cell culture and subsequently in tumor-bearing mice. We initially determined the IC_50_ of both compounds in MC38 (NCB: 0.38 × 10^−6^ m; MBZ: 2.84 × 10^−6^ m) and CT26 cells (NCB: 0.60 × 10^−6^ m; MBZ: 4.29 × 10^−6^ m; Figure S3, Supporting Information). We recognize that the IC_50_ for NCB is higher in MC38 and CT26 compared to the reported IC_50_ in HCT116 cells (21 nM).^[16]^ We presume that the higher IC_50_ in our studies are due to the lower TNIK expression in MC38 and CT29 compared to HCT116.^[16]^ We also realize that the IC_50_ for MBZ is quite high for a kinase inhibitor.

### 2.2. TNIKi Delay Cancer Progression in Preclinical CRC Models

**Figure 1.**
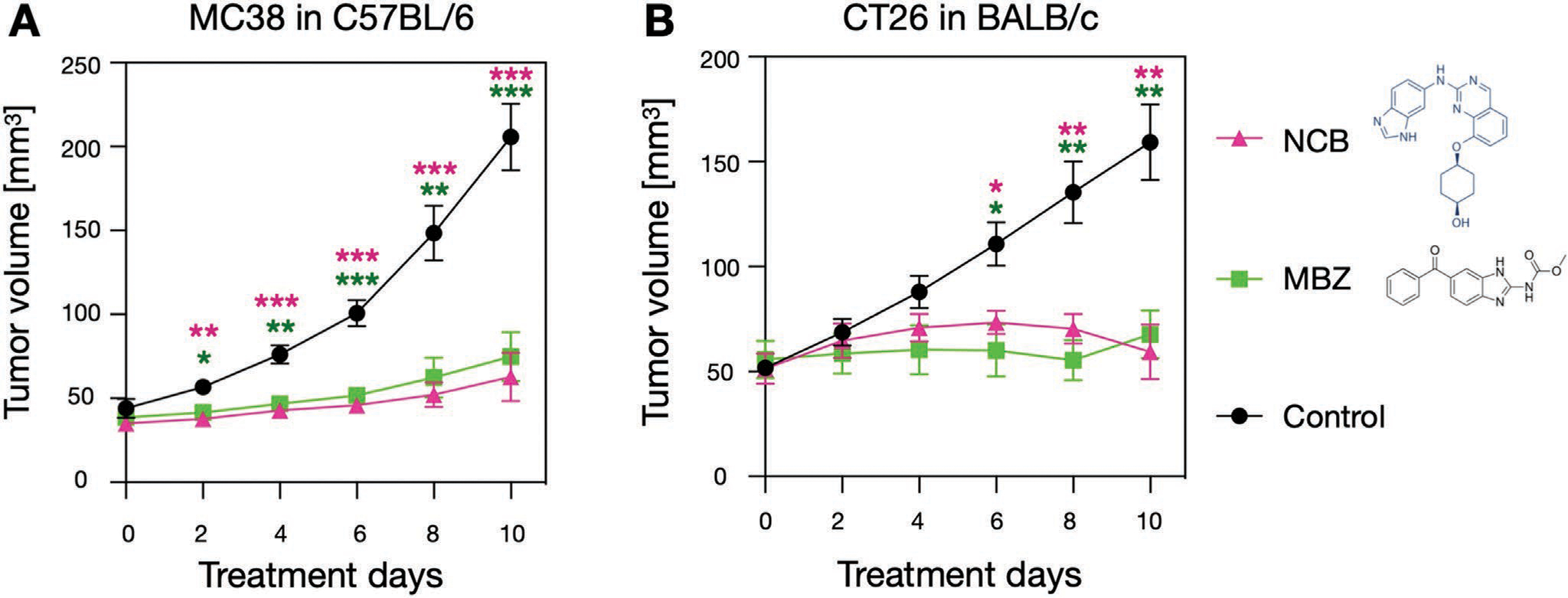
Restrained growth of MC38 and CT26 tumors upon TNIK inhibition. In vivo treatment of NCB0486 or mebendazole in A) MC38 (*n* = 6 per experimental group) and B) CT26 tumor bearing mice (*n* = 6 per experimental group) showed reduction in tumor growth. Both TNIKi were given as monotherapy. Data are presented as mean ± standard deviation. Student’s *t*-test was used for statistical analysis at each time point of tumor size measurement (*<0.05, **<0.005, ***<0.0005).

When applied in cell culture, both TNIKi decreased cellular TNIK and *β*-catenin expression as well as downstream pathway targets including c-MYC, Lrp5/6, and axin2 as measured by flow cytometry (Figure S4, Supporting Information). Given the inhibitory effects, we next tested both compounds for their ability to restrain tumor growth in vivo. Both agents showed efficacy over vehicle control groups. We found that C57BL/6 mice bearing MC38 tumors showed significantly lower tumor burden after 10 days of NCB or MBZ treatment compared to vehicle control group (control vs NCB, *p* < 0.001; control vs MBZ, *p* < 0.001; Figure 1A). Likewise, BALB/c mice bearing CT26 tumors showed significantly lower tumor burden after 9 days of NCB or MBZ treatment compared to the untreated mice (control vs NCB, *p* < 0.005; control vs MBZ, *p* < 0.005; Figure 1B)

### 2.3. TNIKi-Induced Tumor Control Involves Adaptive Immunity

**Figure 2.**
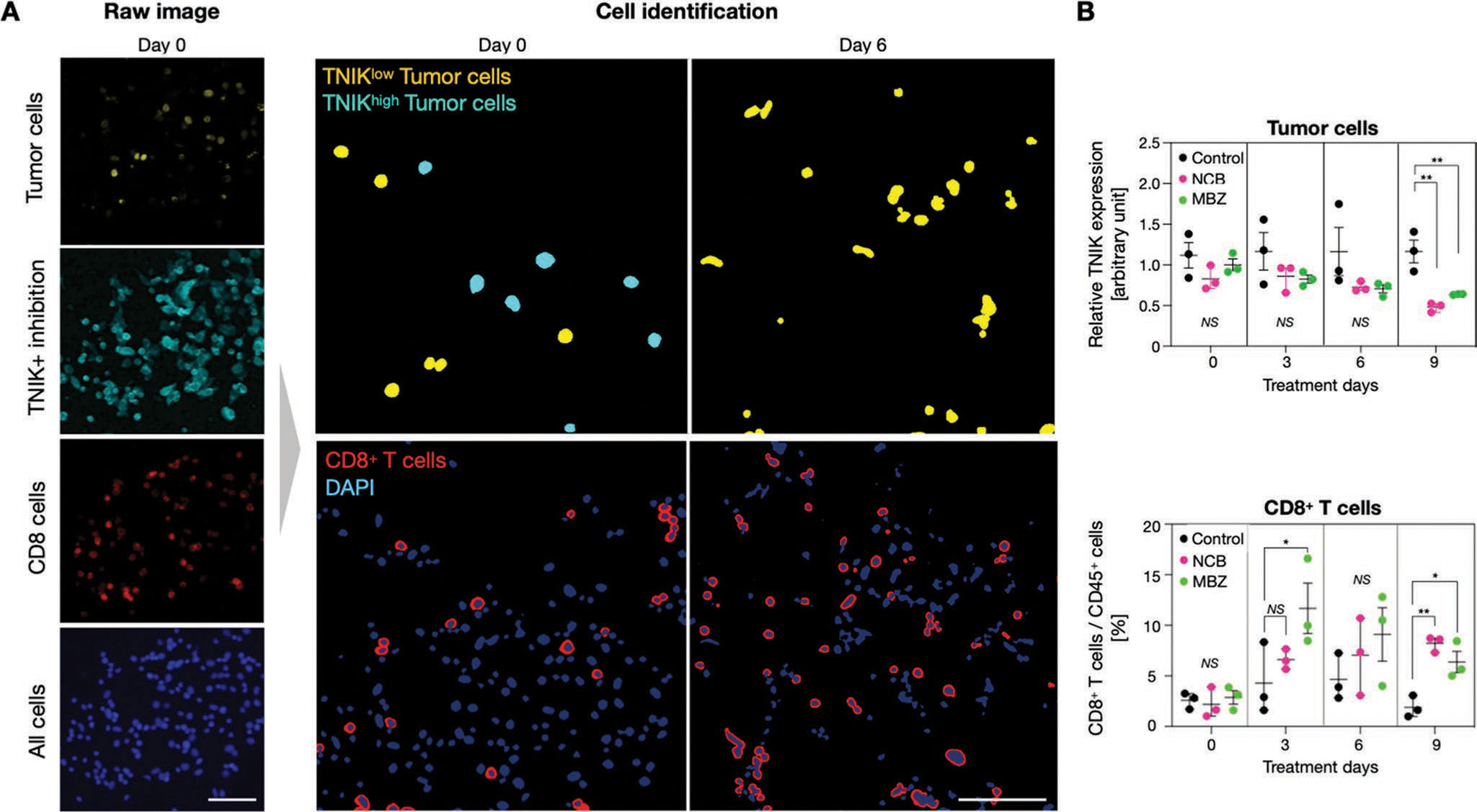
Serial profiling of mouse tumors undergoing TNIKi treatments. Multiplexed cellular analysis of tumor fine needle aspirate (FNA) was performed using FAST imaging (FAST-FNA). Cellular samples were obtained on days 0, 3, 6, and 9 of TNIKi treatment. A) Representative images of tumor cells, TNIK-expressing cells, CD8^+^ cells in a FNA sample and examples of cell identification analysis are shown (scale bar: 100 μm). GFP-expressing MC38 cells were implanted and the tumor cells were detected by FAST-labeled anti-GFP antibody. Cell identification was performed according to the algorithm shown in Figure S6 (Supporting Information). B) TNIK expression level in tumor cells and the frequency of CD8^+^ T-cells were quantified at each time point of FNA collection (*n* = 3 per group). GFP signal was used to identify MC38 tumor cells expressing H2B-GFP. CD8^+^ T-cells were identified by the following maker combination: CD45^+^ CD3^+^ CD8^+^ (detailed cell classification information outlined in Figure S5, Supporting Information). Additional FNA analysis is shown in Figure S7 (Supporting Information). Each data point in (B) is presented with mean ± standard deviation. One-way ANOVA with Dunnett’s multiple comparisons tests were used for statistical analyses (*<0.05, **<0.005, ***<0.0005, NS: not significant).

**Figure 3.**
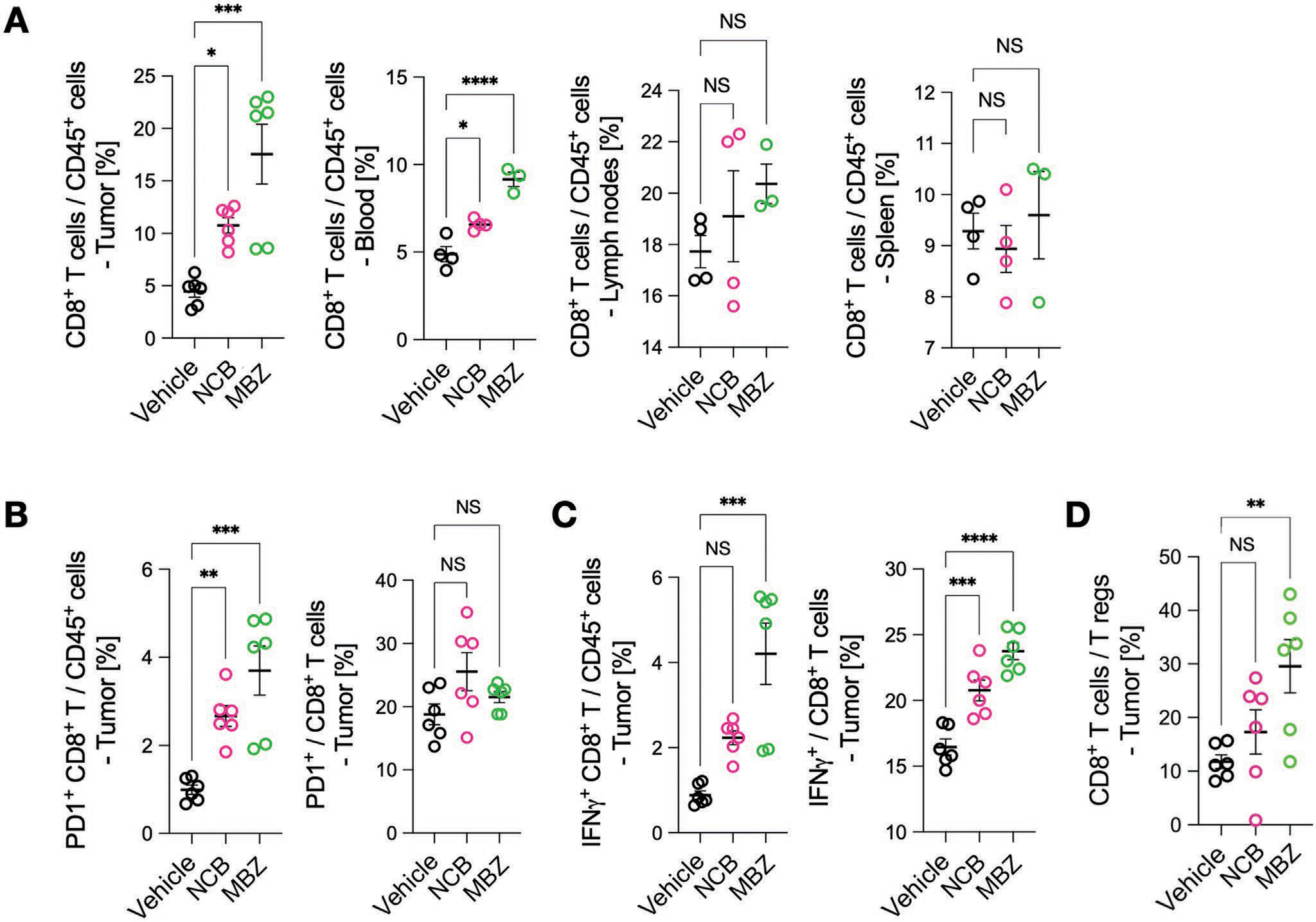
Changes in the intra-tumoral immune phenotype after TNIKi treatment in MC38 tumors. A) Tumors were harvested at day 9 of treatment for flow cytometry analysis for endpoint analyses. With TNIK inhibition in the MC38 model, tumoral infiltration of CD8^+^ T-cells was significantly increased as seen in the serial analysis in Figure 2 and Figure S7 (Supporting Information), whereas such a change was not detected in lymph nodes or spleens of the same tumor-bearing mice. B) The number of PD-1^+^ CD8^+^ T-cell was increased indicating potential up-regulation of immune checkpoint pathway signaling. C) The number of IFN-*γ*^+^ CD8^+^ T-cells was also increased, indicating that a fraction of the CD8^+^ T-cells possess effector function. D) The CD8^+^ T-cell/*T*_reg_ ratio was also increased with TNIK inhibition. Each data point in (B–F) is presented with mean ± standard deviation. One-way ANOVA with Dunnett’s multiple comparisons tests were used for statistical analyses (*n* = 6 per experimental group; *<0.05, **<0.005, ***<0.0005, NS: not significant).

We next performed serial tumor sampling in the same tumors using FAST-FNA to monitor the tumor microenvironment noninvasively during TNIKi therapy (Figures S5 and S6, Supporting Information). FAST-FNA analysis enables multiplexed single cell analysis of tumor-infiltrating immune cells even in scant amount of samples such as FNA and has been thoroughly validated in our previous studies, which profiled various types of immune cells in both mouse and human tumor samples.^[8,17]^ Figure S5 (Supporting Information) outlines a typical FAST-FNA analysis of an FNA sample obtained from MC38 tumor. FAST-FNA analysis on TNIKi-treated tumors surprisingly revealed that the administration of NCB or MBZ substantially increased the number of tumor-infiltrating CD8^+^ T cells at day 9 of treatment and, to a lesser extent, CD4^+^ T cells at earlier time point of TNIKi treatment (Figure 2; Figure S7, Supporting Information). We further investigated PD-1 expression by T cells, considering that it can identify cells with specificity for tumor antigens,^[18]^ as well as IFN-*γ*, considering the relevance of this cytokine in driving antitumor immunity^[19]^ (Figure S7, Supporting Information). We found that PD-1 and IFN-*γ* expression increased with tumor treatment, but with distinct kinetics. Endpoint analysis by flow cytometry at day 9 confirmed the significantly increased infiltration of CD8^+^ T IFN-*γ* in both MC38 and CT26 tumors (Figure 3A–C; Figure S8A–C, Supporting Information). Elevated CD8^+^ T cell infiltration was not observed in lymph nodes or spleens of the same tumor-bearing mice. TNIKi treatment also increased the ratio of CD8^+^ T cells to Treg cells (Figure 3F), which is associated with beneficial outcomes.^[20]^ These data suggest that TNIKi promotes the emergence of an antitumor immune response.

**Figure 4.**
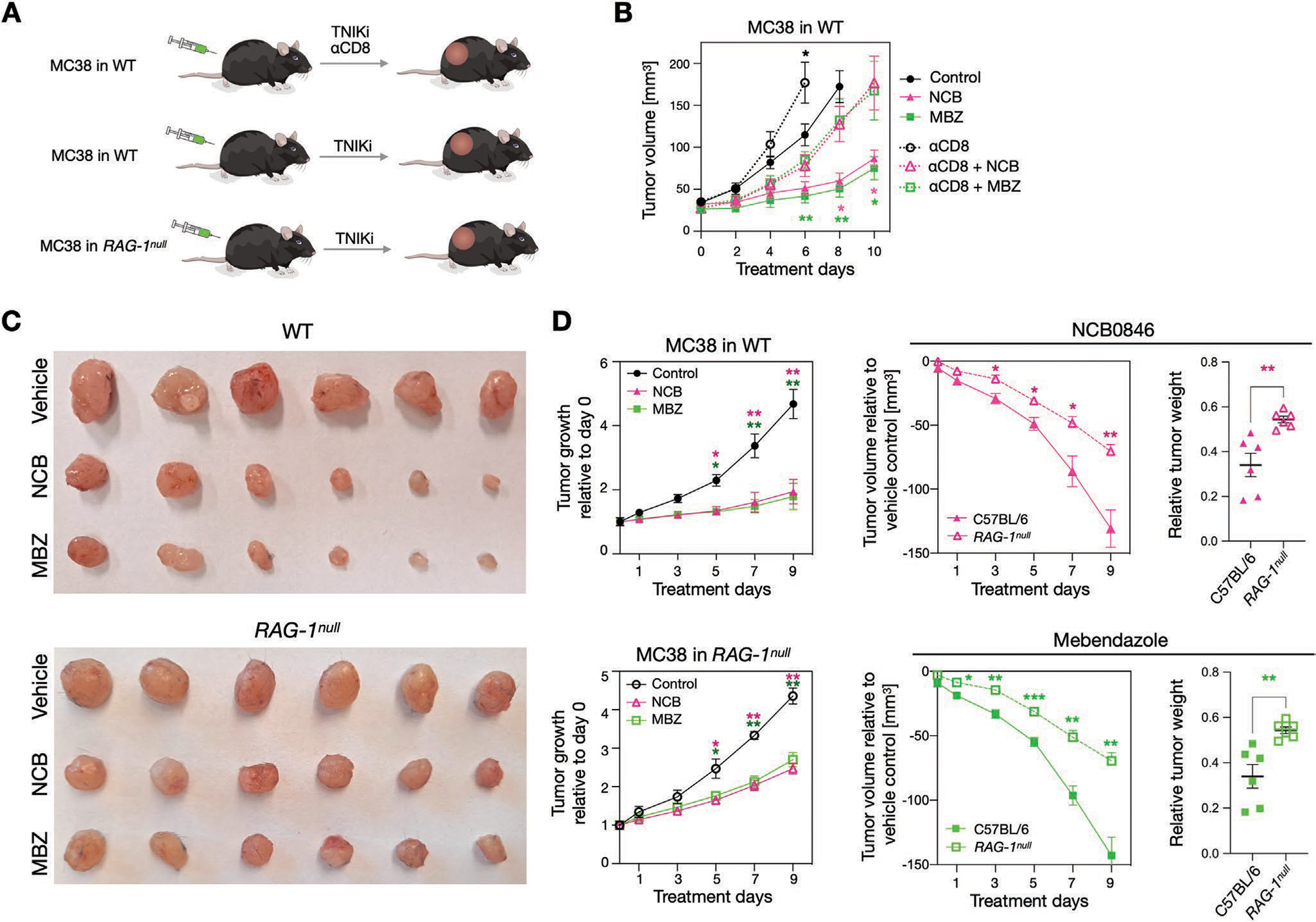
CD8^+^ T-cells play a role in the TNIKi-mediated tumor control. A) To examine the causal effect of CD8^+^ T-cell infiltration on the tumor control of TNIK inhibition, we performed CD8 depletion studies in MC38 tumor-bearing mice. We also monitored the tumor growth in *RAG-1*^null^ mice with treatment of NCB or MBZ to further confirm the involvement of adaptive immune system in TNIKi-mediated tumor control. B) CD8 depletion accelerated the tumor growth of MC38 in mice treated with NCB or MBZ, abrogating the tumor control effect of TNIK inhibition. CD8 depletion also accelerated tumor growth in vehicle control group as expected. Statistical analysis was done between isotype control and CD8 depletion groups for NCB-treated, MBZ-treated, or untreated mice (*n* = 5 per group). C) Images of representative MC38 tumors in immunocompetent WT mice (top) and immunodeficient *RAG-1*^null^ mice (bottom) with or without TNIK inhibition are shown. D) In *RAG-1*^null^ mice, the tumor control of TNIK inhibition was significantly reduced as compared to that in WT mice (*n* = 6 per group). Data are presented as mean ± standard deviation. Student’s t-test was used for statistical analysis at each time point of tumor size measurement (*<0.05, **<0.005, ***<0.0005).

To test the importance of CD8^+^ T cells in the therapeutic control of CRC tumor progression, we performed two additional studies (Figure 4A). First, we depleted CD8^+^ cells in C57BL/6 mice bearing MC38 tumors. Compared to IgG isotype control groups, CD8^+^ cell depletion eliminated much of the efficacy of TNIKi in controlling tumor progression (Figure 4B). Next, we used C57BL/6 *RAG-1*^null^ mice, which lack mature T and B cells. Again, in the *RAG-1*^null^ mice, TNIKi were less effective in controlling MC38 tumor growth compared to WT mice. For example, at day 9, the relative tumor weight for NCB-treated mice was 0.35 mg in the WT and 0.6 mg in the *RAG-1*^null^ model (*p* < 0.005; Figure 4). Similar findings were observed in MBZ-treated animals (Figure 4C,D). These findings indicate that adaptive immune responses are involved in tumor control resulting from TNIK inhibition.

### 2.4. Immunogenic Effects of TNIKi on Tumor Cells

Some drugs targeting tumor cells can cause so-called immunogenic tumor cell death, which triggers powerful downstream antitumor immune responses.^[9,21]^ Since the activation of this process is promising for the treatment of cancer, we tested whether TNIKi treatment can induce immunogenic tumor cell death and thus activate an antitumor immune response.

**Figure 5.**
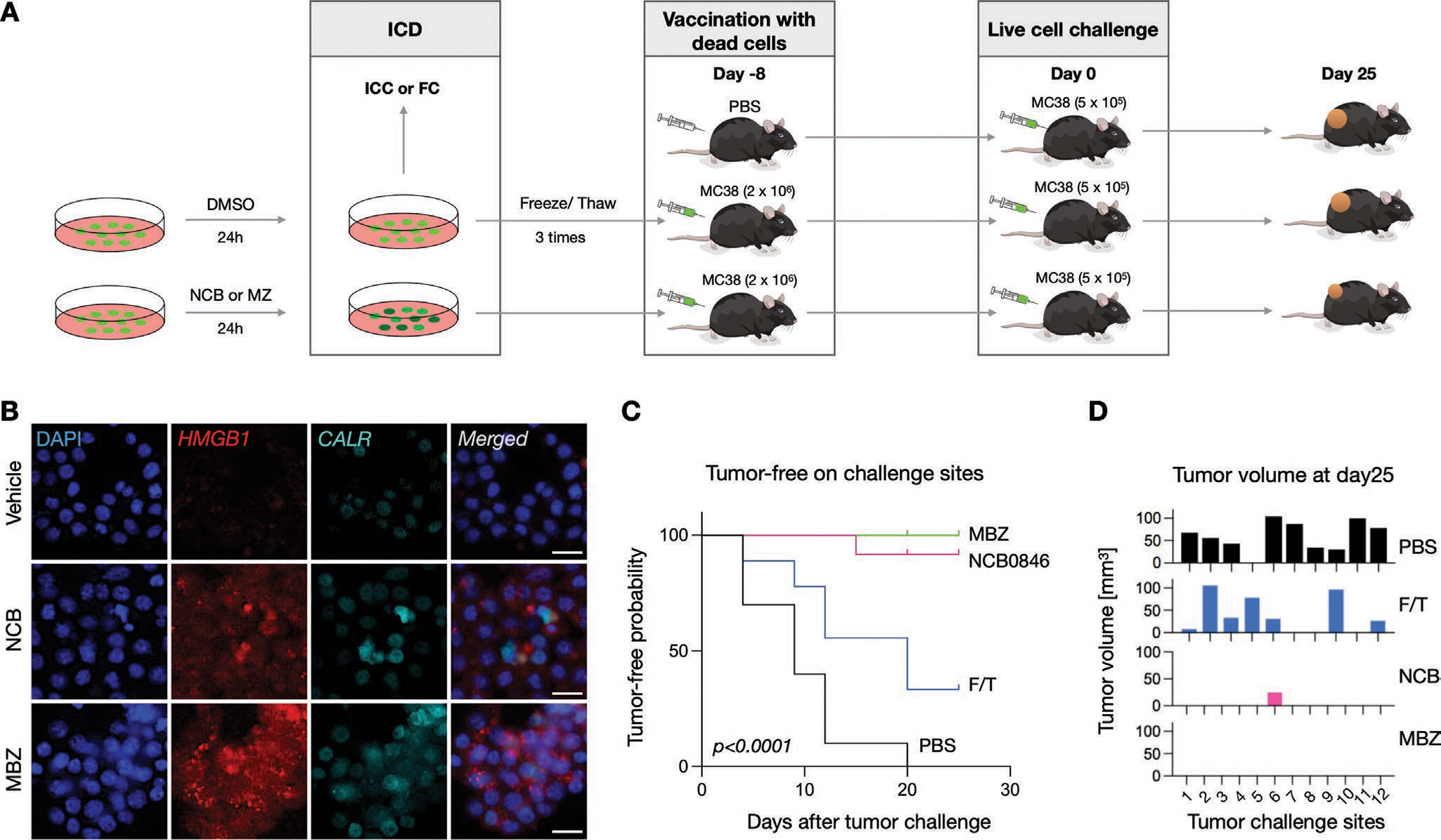
Vaccination with TNIKi-treated cells prevents tumor growth. A) Outline of the experimental scheme. C57BL/6 mice were exposed to MC38 tumor cells that had been killed in vitro by NCB or MBZ. Control mice received PBS or MC38 cells killed in vitro by multiple freeze/thaw (FT) cycles (*n* = 4, 12 tumors per group). Eight days later, these cohorts of mice were all injected with live MC38 cells. B) Tumor cells treated with NCB or MBZ in vitro showed elevated expression of HMGB1 and CALR, markers indicating immunogenic cell death (scale bar: 25 μm). C,D). Tumor-free probability and the tumor volume at day 25 of tumor challenge was quantified in the animal groups that received MBZ-treated cells (green), NCB-treated cells (pink), cells underwent freeze/thaw cycles (blue), or PBS (black). MC38 cells developed much more slowly in mice that had been previously exposed to NCB-killed MC38 cells than the control groups. Likewise, MC38 cells were unable to develop visible tumors in mice previously exposed to MBZ-killed MC38 cells.

First, we investigated whether tumor cells exposed to TNIKi produced markers of immunogenic cell death (Figure 5A). Indeed, we observed that TNIKi treatment of MC38 cells was sufficient to increase the expression of two prototypical immunogenic cell death markers, namely HMGB1 and calreticulin (Figure 5B). Second, we tested whether limiting TNIKi exposure to tumor cells could be sufficient to activate an antitumor response in mice. To this end, C57BL/6 mice were exposed to MC38 tumor cells that had been killed in vitro by NCB or MBZ (Figure 5A). Control mice received MC38 cells killed in vitro by multiple freeze/thaw (FT) cycles. Eight days later, these cohorts of mice were all injected with live MC38 cells. Remarkably, the latter MC38 cells developed tumors much more slowly in mice that had been previously exposed to NCB-killed MC38 cells, as compared to the control groups. Furthermore, these same MC38 cells were unable to develop visible tumors in mice previously exposed to MBZ-killed MC38 cells (Figure 5C,D). These results indicate that TNIKi can promote immunogenic tumor cell death and trigger antitumor adaptive immunity.

We then investigated whether TNIKi treatments trigger its potent CD8^+^ T cell-mediated antitumor responses solely by inducing immunogenic tumor cell death^[9]^ or alternatively by directly activating CD8^+^ T cell. For these experiments, we isolated CD8^+^ T cell from spleen and tumor microenvironment of MC38 tumor bearing C57BL/6 mice. Isolated CD8^+^ T cell was treated with or without TNIKi in culture and then analyzed by flow cytometry for CD25, CD44, CD62L, and CD69 expression at various time points for up to 48 h. Figure S9 (Supporting Information) shows that both drugs can activate CD8^+^ T cell directly with the fraction of CD44^+^CD62L^−^ T cells increasing and that of CD62L^+^CD44^−^ cells decreasing. These phenotypic changes indicate that CD8 cells are activated directly by both of the TNIKi. This suggests that there are at least two ways that the drugs can promote antitumor immunity.

### 2.5. Immunogenic TNIKi Sensitizes CRC to Immune Checkpoint Therapy

**Figure 6.**
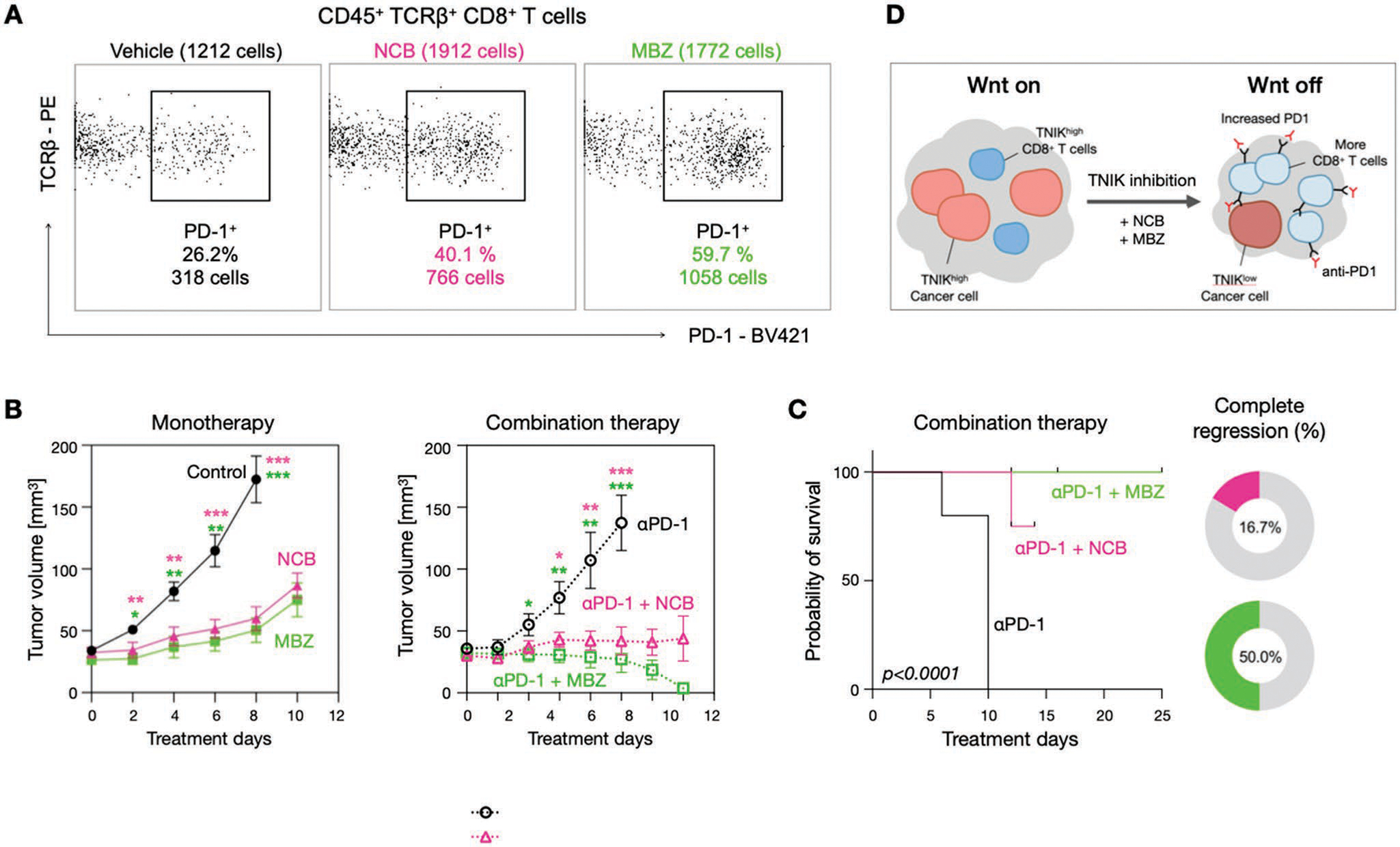
TNIKi-induced CD8^+^ T cell infiltration can potentiate immune checkpoint blockade. A) Flow cytometry analyses indicate that PD-1 level in CD8^+^ T-cells increase with TNIKi treatment providing a rationale for anti-PD1 combination treatment. B) Anti-PD-1 treatment administered in combination with TNIKi (right) resulted in greater tumor regression in a fraction of MC38 mice than TNIKi monotherapy (left) (*n* = 6 per group). C) Working model of the immunogenicity of TNIKi monotherapy and its role in successful TNIKi/ICB combination therapy. Data are presented as mean ± standard deviation. Student’s t-test was used for statistical analysis at each time point of tumor size measurement (*<0.05, **<0.005, ***<0.0005)

Given the fact that TNIKi-treated tumors resulted in marked CD8^+^ T cell infiltration, we next asked whether this process could be harnessed to sensitize tumors to immune checkpoint blockade (ICB) therapy. We were motivated to use anti-PD-1 in particular considering that its target was expressed by the T cells that were accumulating in tumor following TNIKi treatment. Specifically, 59.7% of CD8^+^ T cells were PD-1^+^ in MC38 tumors of MBZ-treated mice and 40.1% were PD-1^+^ in NCB-treated mice, while only 26.2% of the CD8^+^ T cells were PD-1^+^ in the control group (Figure 6A). In tumor growth studies, anti-PD-1 treatment was synergistic to TNIKi treatment resulting in complete regression of tumors in 50% of mice co-treated with MBZ and in 16.7% of the mice treated with NCB (Figure 6B). Either treatment alone showed continuous tumor growth as expected. Together, these findings reveal immunogenic properties of TNIKi (Figure 6C) and suggest that the response rate to checkpoint therapy in CRC could be improved by combining it with immunogenic TNIK kinase inhibitors.

## 3. Discussion

The goal of the current study was to temporally profile the tumor microenvironment in murine CRC undergoing TNIK inhibition. TNIKi have been shown to inhibit tumor growth of Wnt-addicted cancers. We reasoned that treatment efficacy could be quantitated by cellular response markers (TNIK protein levels, cell viability, Wnt downstream targets). While we observed these changes, concomitant analysis of the tumoral immune microenvironment showed a remarkable immune infiltration with CD8^+^ T cells. Since tumoral T lymphocyte infiltration is one of the biomarkers for efficient immunotherapy,^[9]^ we reasoned that TNIKi monotherapy could prime CRC tumors to immune checkpoint inhibitors. We indeed show that the antitumoral effects of TNIKi can be further enhanced by anti-PD-1 treatment, indicating an unexpected immunogenicity mediated by kinase inhibition.

To date few readily druggable targets within the Wnt pathway have been identified. TNIK kinase is essential in the activation of the *β*-catenin pathway and many CRC patients with advanced stages II and III have been shown to have elevated TNIK protein levels.^[22]^ It is recruited to the promoters of the Wnt target genes and directly phosphorylates TCF4.^[23,24]^ However, TNIK is a multifunctional protein,^[25,26]^ and its role is not limited to modulating Wnt signaling. It is also known to regulate stress responses through the c-Jun N-terminal kinase (JNK) pathway,^[26,27]^ cytoskeleton rearrangements^[28]^ and plays roles in the AKT pathway, autophagy, and epithelial–mesenchymal transition.^[29]^ Small molecules targeting TNIK have been reported to suppress tumor initiation,^[5,23,29]^ which has been a major impetus in developing second generation kinase inhibitors.

Numerous small molecule kinase inhibitors are now in clinical use;^[30]^ however, the development of TNIKi remains at an early stage. Small molecule screens^[31]^ and computational approaches^[32]^ have identified a few lead compounds, with other more selective compounds actively being developed. In the current study, we utilized two well-studied model compounds, NCB0846 and mebendazole (MBZ), as prototypical representatives of the class. NCB0846 is a small molecule quinazoline analog. It is orally bioavailable and has been reported to have a half-maximal inhibitory concentration (IC_50_) of 360 × 10^−9^ m in HCT116 cells.^[16]^ In our studies, we found slightly higher IC_50_ values of 380 × 10^−9^ m for MC38 and 600 × 10^−9^ m for CT26 (Figure S4, Supporting Information), presumably because of lower TNIK expression in MC38 and CT26 compared to HCT116.^[16]^ Like many small molecule kinase inhibitors, NCB0846 exhibits good but incomplete selectivity, and the inhibition of FMS-like tyrosine kinase 3 (FLT3), platelet derived growth factor-a (PDGFRa) and cyclin-dependent kinase 2 (CDK2)/cyclin A2 (CycA2) has also been reported. In our study, we confirm that NCB0846 reduces the expression of TNIK at the protein level as well as the Wnt target genes AXIN2 and MYC. Mebendazole is an FDA-approved anti-helminthic in clinical use, exploiting its direct effects on parasite tubulin dimerization, mitotic spindle formation and apoptosis.^[33]^ It has also recently been shown to inhibit TNIK kinase activity^[32]^ in addition to other kinases such as MAPK14 (p38a).^[34]^ The effects observed in our study are thus likely attributable to multiple functions of this drug not just inhibition of TNIK.

Our results indicate that both compounds robustly inhibit TNIK in tumor cells and lead to CD8^+^ T cell infiltration. Currently available TNIKi likely exert their effects on multiple cell types, particularly in those whose TNIK levels are elevated. Interestingly, TNIK is also moderately expressed in T-cells and some effects have been reported in inflammatory disease models. Indeed, the role of TNIK in modulating immune cells either by affecting *β*-catenin transcription in T-cell differentiation^[6]^ or via the canonical NF-*κ*B and c-Jun N-terminal kinase (JNK) activation in B-cells have been reported.^[26]^ Our in vitro experiment shows that both NCB0846 and MBZ can directly activate CD8^+^ T-cells independently of immunogenic tumor cell death. We therefore conclude that the antitumor effect of TNIKi is due to two nonmutually exclusive possibilities: i) tumor control by immunogenic tumor cell death and ii) direct drug effects on CD8^+^ T-cells.

Overall, our findings provide insight into the immunogenic nature of TNIK inhibition in Wnt-addicted cancers, which can motivate future clinical trials, especially with FDA-approved TNIKi, such as mebendazole, in combination with checkpoint inhibitors and/or other immunotherapies.

## 4. Experimentsal Section

### Materials:

Vendors and catalog numbers of the antibodies used for immunoprofiling are summarized in Table S1 (Supporting Information). All antibodies were tested and validated on positive cell lines or mouse splenocytes for validation before usage. Anti-PD-1 antibody was purchased from Bio X Cell (Clone: 29F.1A12) for the combination treatment with TNIKi. NCB0846 was obtained from Selleckchem (S8392) and mebendazole was obtained from Selleckchem (S4610) or Sigma–Aldrich (M2523).

### Cell Lines:

The MC38 cell line was a kind gift from Mark Smyth (QIMR Berghofer Medical Research Institute). The CT26 cell line was purchased from ATCC. For detection of tumor cells for imaging and flow cytometry, MC38-H2B-GFP and CT26-H2B-GFP cell lines were generated by viral transfection. Cells were cultured in IMDM medium (Gibco) with 10% FBS and 1% penicillin/streptomycin. MC38 and CT26 were two of the most commonly used mouse syngeneic CRC cell lines in preclinical studies of colorectal cancer. CT26 were reported previously to be pMMR CRC cell lines^[12]^ while MC38 was mismatch repair-deficient dMMR.^[13]^ Both models generally recapitulated response to immunotherapy^[35]^ when implanted orthotopically.^[13]^ These model murine model lines were well established^[36]^ and were used in many immunologic tumor studies.

### Mouse Studies:

WT C57BL/6, *RAG-l*^null^, and WT BALB/c mice with 8 weeks of age were purchased from Jackson Laboratory. MC38 or CT26 (2 × 10^6^ cells per 50 μLsterile PBS per implant) was injected subcutaneously for tumor implantation. All animals were housed under specific pathogen free conditions at the Massachusetts General Hospital. Experiments were approved by the MGH Institutional Animal Care and Use Committee (IACUC) and were performed in accordance with MGH IACUC regulations. For in vivo inhibition of TNIK, NCB0846 was dissolved in DMSO/polyethylene glycol#400/30% 2-hydroxypropyl-b-cyclodextrin solution (10:45:45 by volume) and was administered daily at 50 mg kg^−1^ (b.i.d) by intraperitoneal injection. Mebendazole was prepared in 1:1 mixture of PBS and sesame oil and administered daily by oral gavage at 100 mg kg^−1^. For immunomodulation or cell depletion, anti-CD8a (BE0061, clone 2.43, BioXcell) or anti-PD-1 (BE0273, clone 29F.1A12, BioXcell) was injected every other day at 100 μg per mouse in 0.1 mL sterile PBS. Experiments were approved by the MGH Institutional Animal Care and Use Committee (IACUC) and were performed in accordance with MGH IACUC regulations.

### Antibody Modifications for FAST Imaging:

Carrier-free antibodies were purchased (Table S1, Supporting Information) and conjugated with FAST probes as previously described.^[17]^ Antibodies were buffer-switched into bicarbonate buffer (pH 8.4) using a 40k zeba column (Thermo Fisher). After buffer exchange, antibodies (1–2 mg mL^−1^) were incubated with a five- to tenfold molar excess of the FAST probes with 10% DMSO for 30 min at room temperature. After the conjugation reaction, unbound FAST probes were removed by 40k zeba column equilibrated with PBS. Antibodies conjugated to the FAST probes were stored at 4 °C, protected from light. The degree of labeling (DOL) was determined by measuring the absorbance spectrum of the FAST-labeled antibody using a Nanodrop 1000 (Thermo Scientific). Known extinction coefficients of the specific dye (AF488, AF55, or AF647) and IgG antibody and correction factor for the dye absorbance at 280 nm were applied for DOL calculation.

### Synthesis of Fluorochrome/Quencher Pair:

FAST probes were constructed as a modular linker to connect fluorochromes and antibodies with an embedded TCO that clicked with a tetrazine-quencher. FAST probes were synthesized as previously described, stored as the carboxylic acids, and then custom-activated for antibody labeling with our in situ NHS activation chemistry.^[17]^ The dTCO-PEG_6_-CO_2_H blocking reagent was synthesized in one step from dTCO-PNP and commercially available amino-dPEG_6_-CO_2_H, then characterized by LC-MS. All reagents were obtained from commercial sources at the highest grade available. Fluorophores were purchased from Fluoroprobes or Click Chemistry Tools. BHQ-3 Amine was procured in 5 or 25 mg aliquots from LGC Biosearch Technologies. N-*α*-Boc-N-*ε*-Fmoc-L-Lysine (≥99%) was purchased from Chem–Impex. Amino-dPEG_n_-carboxylic acids (*n* = 4,6) were sourced from Quanta BioDesign. Dry solvents and coupling reagents were from Sigma–Aldrich.

### Immunostaining and Quenching for FAST Imaging:

Cells obtained from fine needle aspiration were attached with a glass slide by cytospin, fixed with 4% PFA for 10 min, and permeabilized with 0.5% Triton-X100 for 25 min in blocking buffer before the first staining. Immuno-labeling for FAST imaging was performed as in typical immunofluorescence protocols and similar to the prior studies.^[8,17]^ Cells were incubated with antibodies in blocking buffer (Intercept, LI-COR Biosciences). Stained cells were washed with PBS three times, 5 min each, and imaged. After the image acquisition, cells were briefly incubated with Tz-BHQ (10 × 10^−6^ m) in PBS-bicarbonate buffer (pH 9) to quench the fluorescence signal. Residual Tz-BHQ was removed by three washes with PBS-bicarbonate buffer, and the same fields of view were imaged to record the quenched signal for background subtraction from the subsequent cycle. Before staining with the next set of FAST antibodies, cells were briefly incubated with 20 × 10^−6^ m dTCO-PEG_6_-CO_2_H to block any residual Tz-BHQ3 from reacting with FAST antibodies of the next cycle. The procedure was repeated until all target proteins were imaged. As a negative control, a fraction of each sample was set aside, incubated with isotype control antibodies, and imaged every cycle following the same protocol as described above.

### Fluorescence Imaging:

Olympus BX-63 upright automated epifluorescence microscope was used for immunofluorescence. DAPI, FITC, Cy3, and Cy5 filters were used to acquire the images of DAPI nuclear stains, AF488, AF555, and AF647 respectively. Depending on the cell density, 15–30 fields of view were imaged for each sample to capture a sufficient number of cells for analysis. Multi Dimensional Acquisition in Metamorph software was used to save *X-Y* coordinate of each field of view to image the same set of cells rapidly in every cycle.

### Image Analysis:

As outlined in Figure S5 (Supporting Information), acquired images were aligned, background subtracted, and cells were segmented in each field of view. Segmented cells were phenotyped using Python 3.7.0 and Cell Profiler 3.1.9. To correct for pixel translations that occur during imaging, images were aligned using cross-correlation in Fourier space.^[37]^ Nuclei and cells were segmented from DAPI and the maximum-intensity-profile of all markers imaged.^[37,38]^ Cells with no nucleus or more than one nucleus and a nuclear-to-cell ratio greater than one were excluded from analysis. Quenched images from the previous cycle were subtracted from the stained image for background correction. For each identified cell, the average fluorescence intensity of each protein marker was calculated. Cells were classified by manual thresholding of average fluorescent intensity of tumor and immune markers. Cells that were positive for mutually exclusive markers were excluded from analysis.

### Flow Cytometry:

Tumors were harvested at the end of longitudinal experiments and minced with scissors for digestion for single cell isolation. Minced tissue was incubated in digestion medium containing 10 U mL^−1^ Collagenase I, 400 U mL^−1^ Collagenase IV and 30 U mL^−1^ DNAse I for 25 min at 37 °C. Then the digestion medium was neutralized by RPMI1640, and digested tissues were further ground by a syringe plunger, and filtered by 70 × 10^−6^ m cell strainer. Blood samples were collected in EDTA-coated tubes to prevent hemagglutination, and were incubated with ACK lysis buffer for 3 min on ice. The cells were washed with PBS, fixed in 4% PFA for 10 min, and washed again with PBS for immunostaining. The cells were incubated with Fc block (Biolegend) before staining with surface antibodies. For intracellular markers, after cells were fixed and permeabilized after surface marker labeling and stained for appropriate antibodies. The samples were washed, filtered, and analyzed by an LSRII flow cytometer.

### Immunogenic Cell Death Induction and Tumor Cell Vaccination:

MC38 cells were treated with NCB0846 (5 × 10^−6^ m) or MBZ (30 × 10^−6^ m) for 12 h in vitro. Immunostaining against HMGB1 and CALR was then performed. The cells treated with TNIKi were washed with PBS, trypsinized, and were injected at the side flanks (2 × 10^6^ cells per 50 μL PBS per mouse) for immune stimulation. For the negative control, PBS (50 μL per mouse) or MC38 cells that were subjected to three times of freeze/thaw cycles (5 min for each step) were injected into flanks (2 × 10^6^ cells/50 μL PBS per mouse). Eight days after injection, untreated MC38 cells (5 × 10^5^ cells/50 μL PBS) were injected at different sites of flanks of the mice for tumor implantation. The growth was monitored every 4—5 days for 25 days following the re-challenge.

### Statistics:

Results were presented as mean ± SEM. Statistical tests included one-way ANOVA followed by Tukey’s or Dunnett’s multiple comparison test. When applicable, the unpaired one-tailed and two-tailed Student’s *t* tests using Welch’s correction for unequal variances were used. Comparison of survival curves was performed with the Log-rank Mantel-Cox test. *p* values of 0.05 or less were considered to denote significance (*p < 0.05; **p < 0.01; ***p < 0.001; ****p < 0.0001; NS, not significant).

## Supplementary Material

supinfo

## Data Availability

The data that support the findings of this study are available from the corresponding author upon reasonable request.
