# Supplementary material for: TNIK Inhibition Has Dual Synergistic Effects on Tumor and Associated Immune Cells
Source: Adv Biol (Weinh). Author manuscript; Available in PMC 2023 Aug 1. (PMC9398996; doi:10.1002/adbi.202200030)
Supplement: supinfo [file NIHMS1815157-supplement-supinfo.docx]

**Supplemental information**

**TNIK inhibition has dual synergistic effects on tumor and associated immune cells**

Jaehee Kim^1#^, Juhyun Oh^1#^, Hannah Peterson^1^, Jonathan C.T. Carlson^1,2^, Mikael J. Pittet^1,3,4^, Ralph Weissleder^1,2,5*^

^1^ Center for Systems Biology, Massachusetts General Hospital, 185 Cambridge St, CPZN 5206, Boston, MA 02114

^2^ MGH Cancer Center, Massachusetts General Hospital and Harvard Medical School, Boston, MA 02114

^3^ Department of Pathology and Immunology, University of Geneva, Agora Cancer Centre, Rue du Bugnon 25A, 1000 Lausanne, Switzerland

^4^ Ludwig Institute for Cancer Research, Lausanne, Switzerland

^5^ Department of Systems Biology, Harvard Medical School, 200 Longwood Ave, Boston, MA 02115

^#^ These authors contributed equally.

*R. Weissleder, MD, PhD

Center for Systems Biology

Massachusetts General Hospital

185 Cambridge St, CPZN 5206

Boston, MA, 02114

617-726-8226

[rweissleder@mgh.harvard.edu](mailto:weissleder@helix.mgh.harvard.edu)

**Figure S1. Baseline measurement of TNIK expression and CD8^+^ T cell infiltration in MC38 tumors. A.** Representative H&E and immunofluorescence images of TNIK and CD8 in frozen tissue sections of a MC38 tumor without any treatment are shown (scale bar: 100µm). Majority of the tumor cells stained positive for TNIK, where as CD8^+^ cells were scarce. CD8 are scarce **B.** The number of tumor-infiltrating T cells (CD8^+^, CD4^+^, and regulatory T cells) per mm^3^ of tumor was quantified by flow cytometry analysis at day 7 and 14 of tumor growth. Each data point in **C** is presented with mean ± standard deviation. Student’s t-test was used for statistical analysis at each time point of tumor size measurement (ns: not significant).


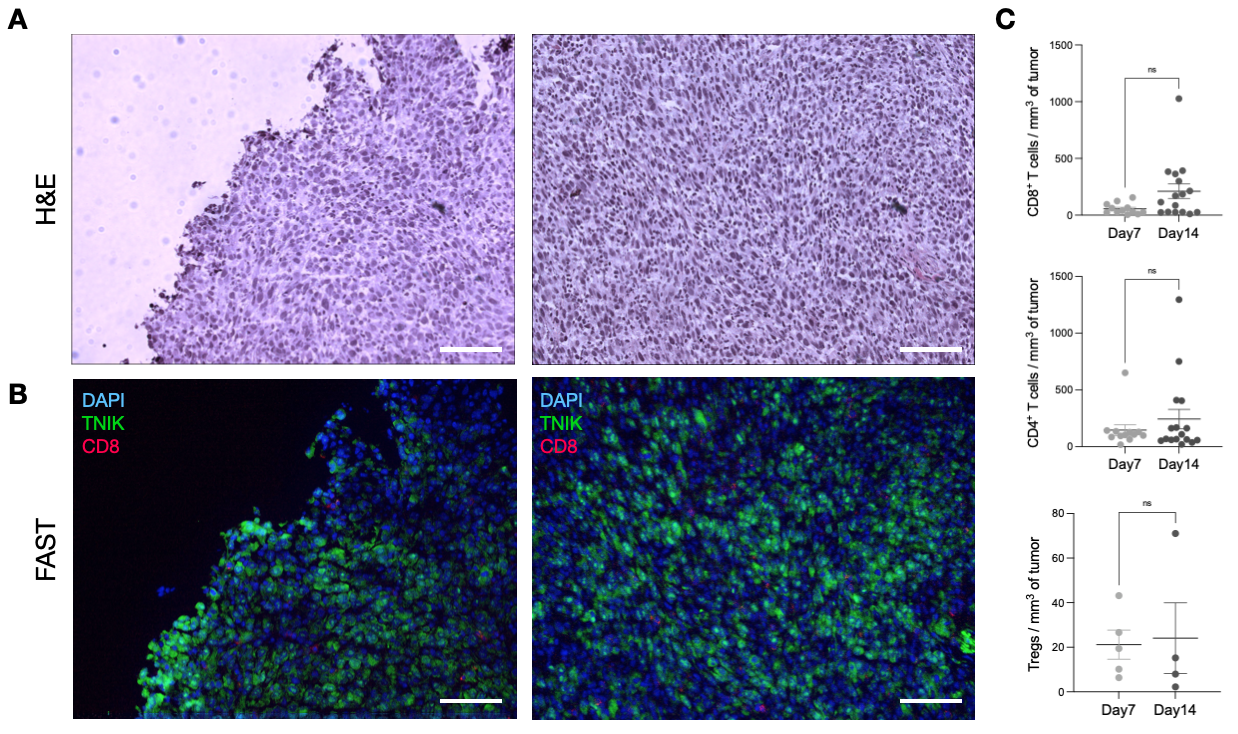


**Figure S2. Study overview**. The effects of the TNIK inhibitors NCB0846 and MBZ were tested in colorectal cancer cell lines MC38 and CT26 *in vitro* and *in vivo*. At day 0, 3, 6, 9 of TNIKi treatment on tumor-bearing mice, FAST-FNA assays were performed to analyze the drug efficiency and immune composition in the tumors. At day 9, tumors were harvested for endpoint analysis by immunofluorescence (IF) and flow cytometry **
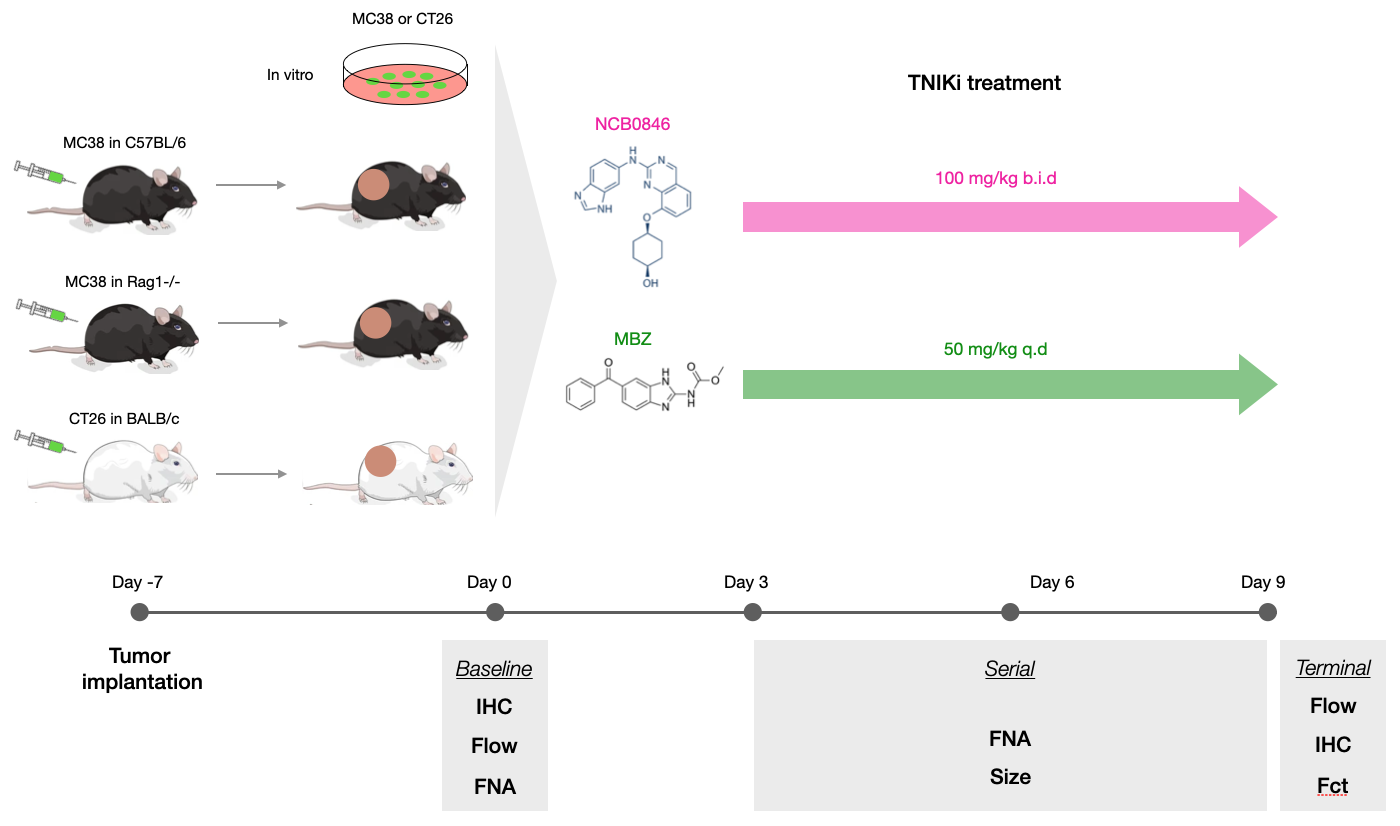
**(Flow). Functional cell analysis (Fct) was performed after 12 hrs of *in vitro* treatment.

**Figure S3. IC_50_ Determination of NCB0846 and MBZ in MC38 and CT26 cells.** In MC38, the IC_50_ was 0.38µM and that of MBZ was 2.84 µM. These values are higher than this reported in the literature for HCT116 cells (0.36 µM for NCB and < 5µM for
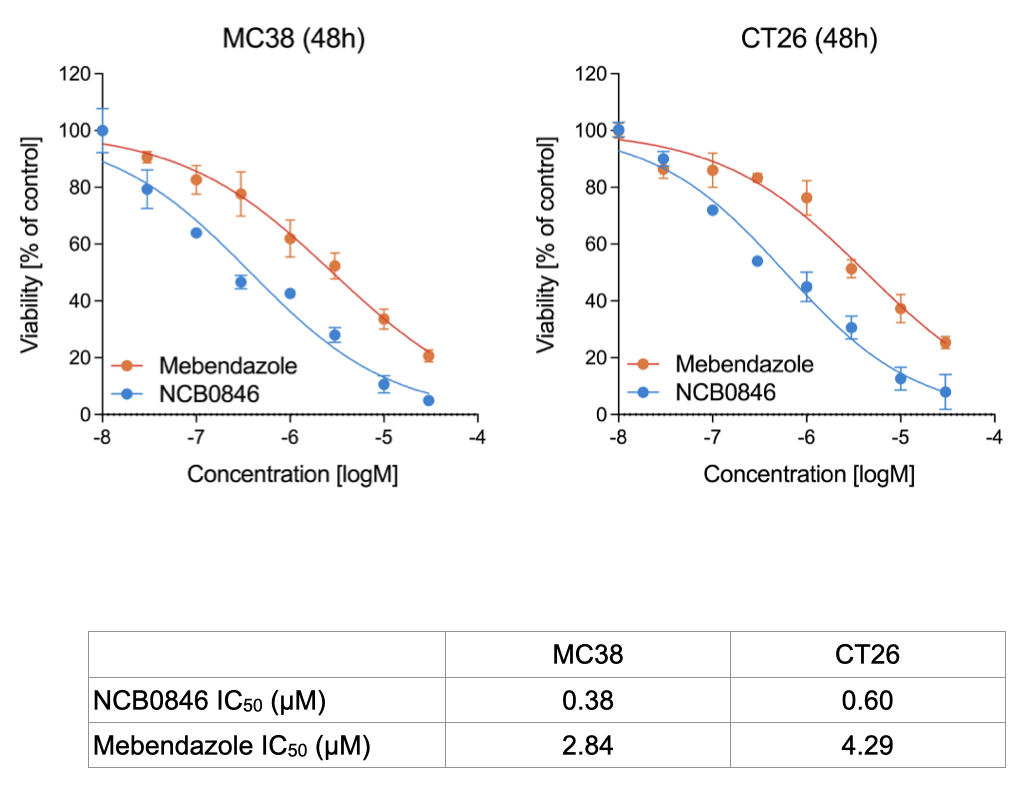
MBZ), presumably due to differences in TNIK concentration between the cell lines.

**Figure S4. Phenotypic changes in MC38 and CT26 tumors upon TNIK inhibition. A.** The role of TNIK in as a regulatory component of the β-catenin pathway is exhibited in the diagram. Wnt transducers and TNIK downstream factors whose expression level was m
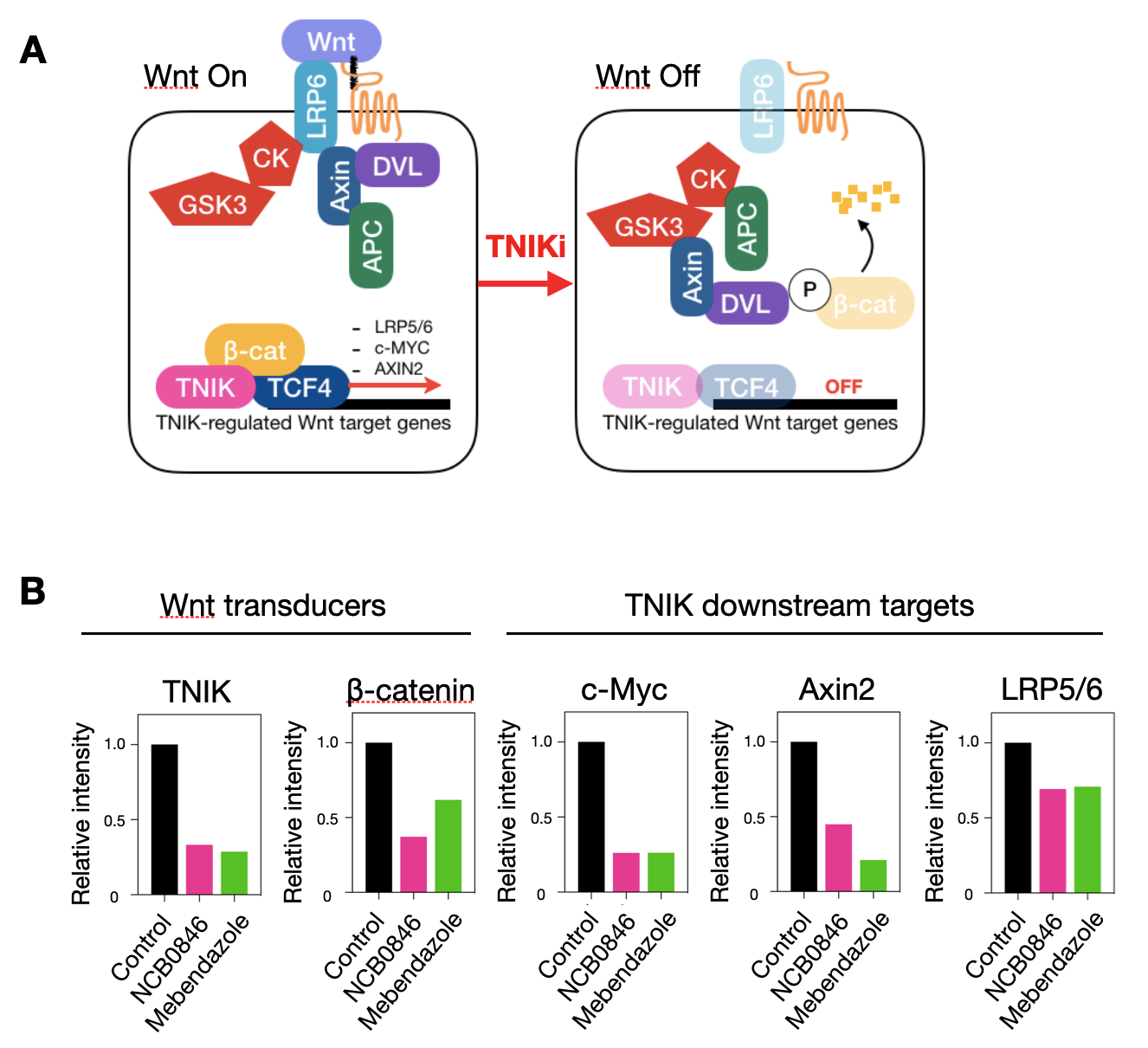
easured in **Figure 1A** are shown. **B.** When MC38 cells were incubated with NCB (5µM) or MBZ (30µM) for 12 hours, inhibitory effects was identified by decreased expression of TNIK, cMyc, axin-2 and LRP5/6 protein by flow cytometry (see **Figure S3** for IC_50_).

**Figure S5. Immune profile of MC38 tumors. A.** Example images of FAST analysis of aspirated cells (scale bar: 25µm). **B.** An example of quantitative analysis of FAST-FNA assay. The fre
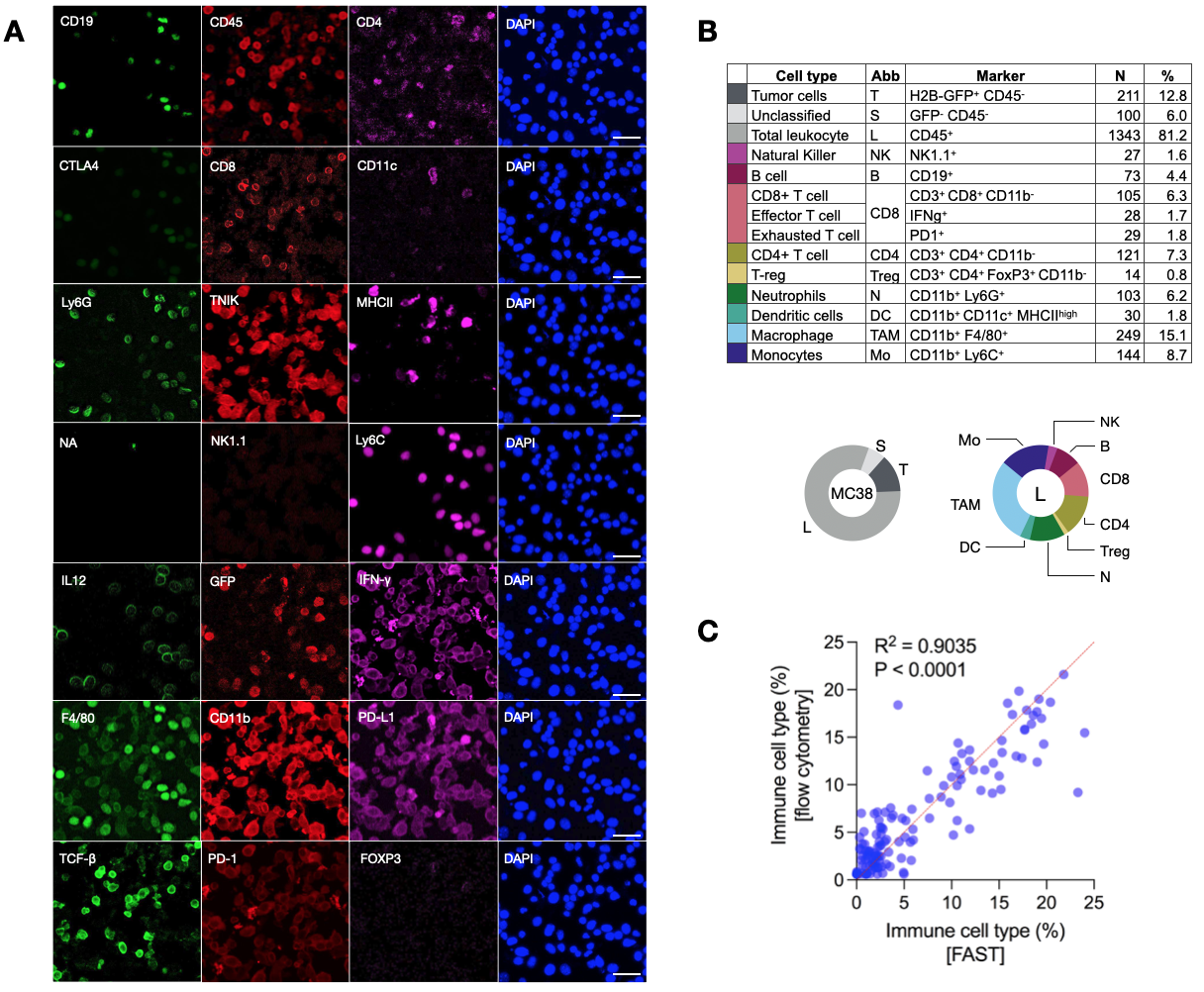
quency of different immune cell types and their subtypes are quantified in each FNA sample. **C.** Correlation between FAST-FNA analysis and flow cytometry analysis of the whole tumor.

**Figure S6. Image analysis pipeline**. **A.** Flow chart of image analysis workflow. **B.** Processed images of tumor FNA on days 0 and 6 showing TNIK-expressing cells and CD8^+^ T cells. Cells of interest are highlighted in red (CD8^+^), cyan (TNIK-high tumor cells), yellow (TNIK-low tumor), and blue (nucleus). The images show TNIK-high tumor cells on Day 0, whose TNIK level significantly reduced on day 6. The number of CD8^+^ T **
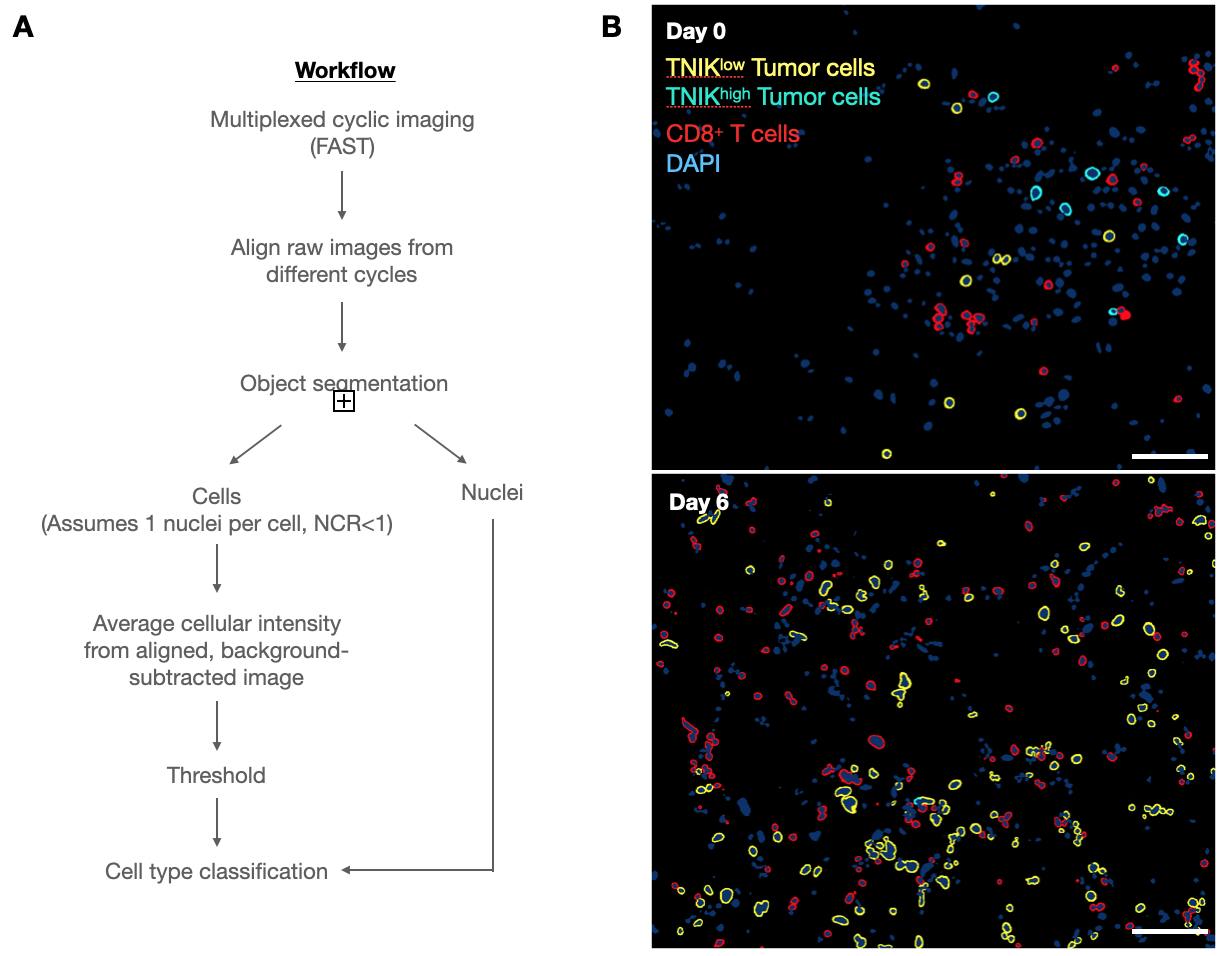
**cells increases over time.

**Figure S7. Serial FAST-FNA analysis of tumor-infiltrating CD8^+^ T cells.** TNIK inhibition by NCB0846 increases CD8^+^T cell infiltration in MC38 tumors over 9 days of treatment. IFNγ^+^ CD8^+^ T cell frequency was elevated at earlier time point and maintained, whereas PD-1^+^ CD8^+^T cell frequency gradually increased until day 6. CD4^+^
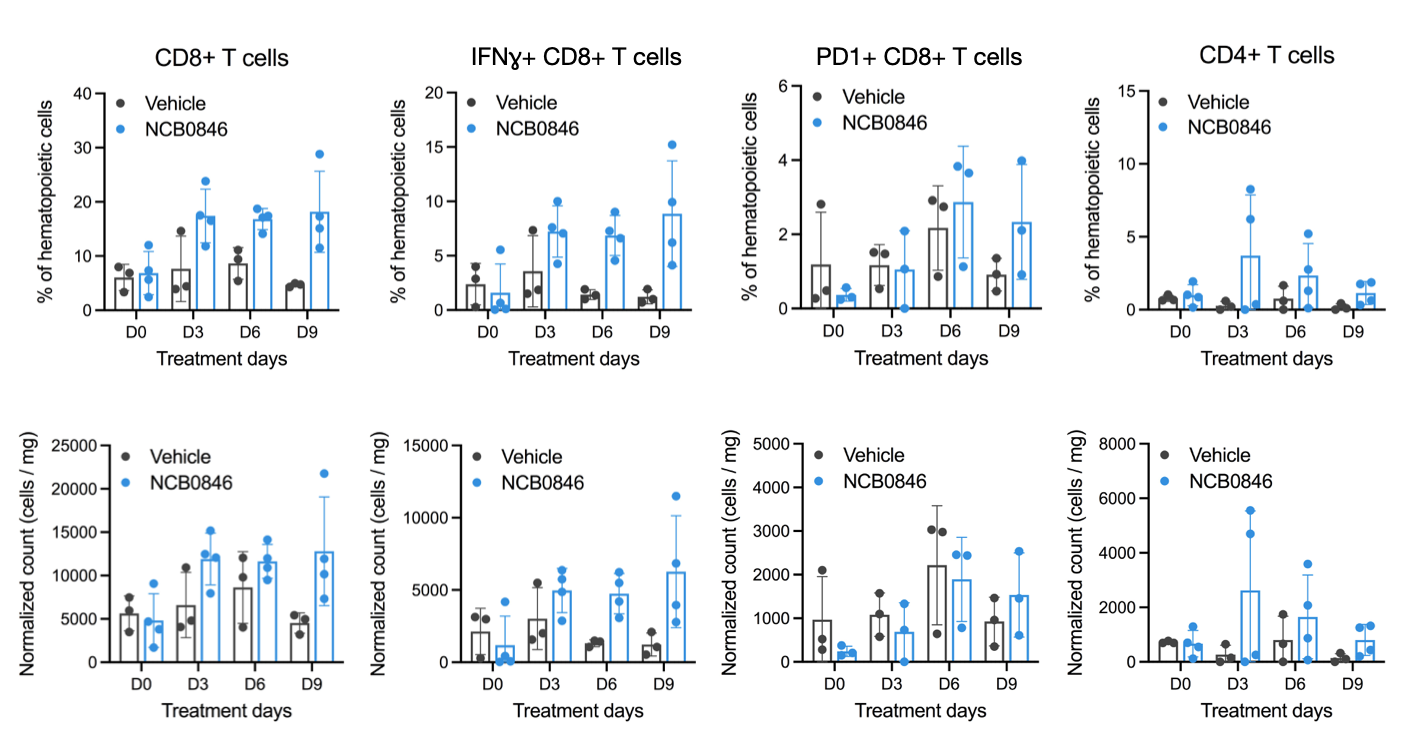
T cell levels did not show meaningful temporal changes.

**Figure S8. Changes in the intra-tumoral immune phenotype after TNIKi treatment in CT26 tumors.** Tumors were harvested at day 9 of TNIKi treatment for endpoint analysis by flow cytometry. **A**. Infiltration of CD8^+^ T cells was significantly increased in CT26 tumors upon TNIK inhibition, whereas such a change was not detected in blood, lymph nodes, or spleens of the same mice. The frequency of PD-1^+^ CD8^+^ T cells (**B**) and IFN-ɣ^+^ CD8^+^ T cells (**C**) was increased with TNIK inhibition as observed in M
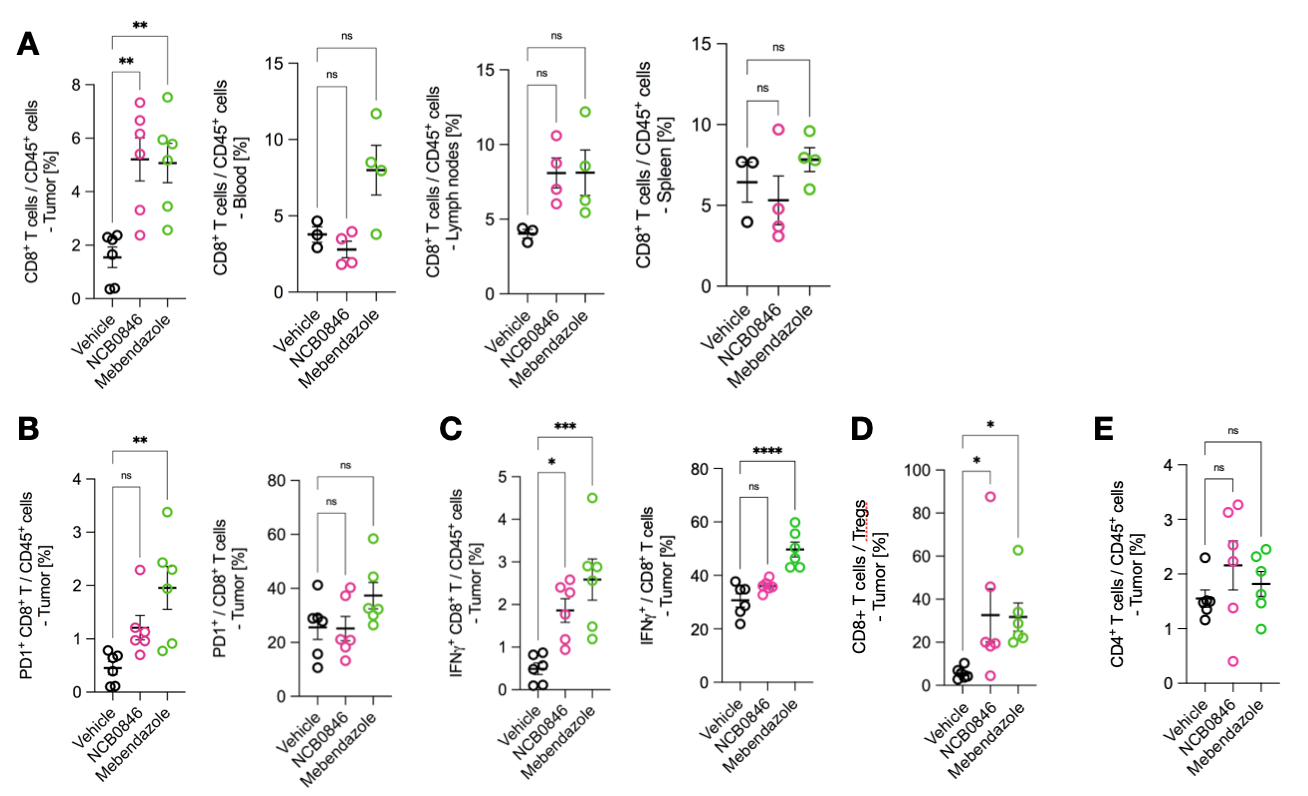
C38 tumors. **D.** CD8^+^ T cell / Treg ratio was also significantly increased with TNIK inhibitor treatment by both drugs. **E**. Infiltration of CD4^+^ T cells didn’t show any significant change with the TNIKi. In general, similar trends as in MC38 tumors were observed (**Figure 3**). Each data point is presented with mean ± standard deviation. One-way ANOVA with Dunnett’s multiple comparisons tests were used for statistical analyses (*<0.05, **<0.005, ***<0.0005, ****<0.00005, ns: not significant).

**Figure S9. Direct effects of TNIKi on CD8^+^ T cells**. CD8^+^ T cells were harvested from spleens of C57BL/6 mice and treated with either NCB0846, mebendazole or vehicle (control). Flow cytometry was performed at 6, 12, 24 and 48 hours following drug exposure. Analysis of CD25, CD44, CD62L, and CD69 level indicated that both drugs **
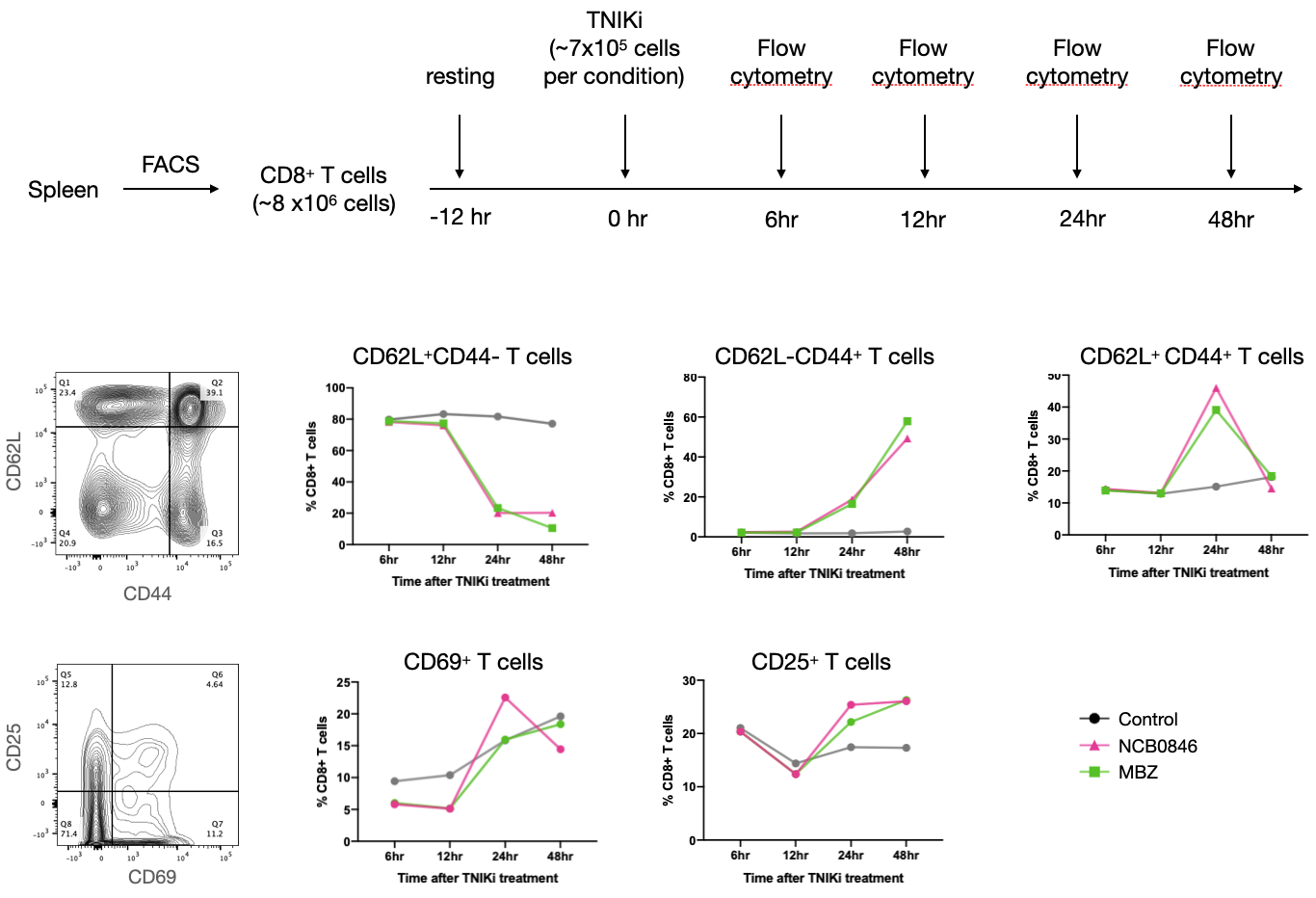
**are capable of activateing CD8^+^ T cells independent of immunogenic tumor cell death.

## Table S1. Antibodies used for serial FAST-FNA profiling of the tumor microenvironment

| **Markers** | **Target population** | **Clone** | **Vendor** | **Catalog #** | **Fluorochrome** |
| --- | --- | --- | --- | --- | --- |
| CD45 | Hematopoietic cells | 104.2 | Bio X Cell | BE0300 | AF488 |
| TCRb | T cells | H57-597 | Biolegend | 109215 | AF488 |
| CD8 | CD8+ T cells | 53-6.7 | Bio X Cell | BE0004-1 | AF555 |
| CD4 | CD4+ T cells | GK1.5 | Bio X Cell | BE0003-1 | AF647 |
| FoxP3 | Regulatory T cells | MF-14 | Biolegend | 126402 | AF647 |
| NK1.1 | NK cells | PK136 | BD Pharmingen | 553162 | AF555 |
| CD19 | B cells | 1D3 | BD Pharmingen | 5378302 | AF488 |
| CD11b | Myeloid cells | M1/70 | Bio X Cell | BE0007 | AF647 |
| F4/80 | Macrophages | CI:A3-1 | Bio X Cell | BE0206 | AF555 |
| CD11c | Dendritic cells | N418 | Biolegend | 117302 | AF647 |
| Ly6G | Neutrophils | 1A8 | Bio X Cell | BE0075-1 | AF647 |
| Ly6C | Monocytes | Monts1 | Bio X Cell | BE0203 | AF488 |
| MHCII | Dendritic cells | M5/114 | Bio X Cell | BE0108 | AF488 |
| PD-1 | Various | 29F.1A12 | Bio X Cell | BE0273 | AF555 |
| PD-L1 | Various | 10F.9G2 | Bio X Cell | BE0101 | AF555 |
| Granzyme B | Cytotoxic T cells | QA16A02 | Biolegend | 372202 | AF488 |
| IFN-ɣ | Cytotoxic T cells | XMG1.2 | Bio X Cell | BE0055 | AF555 |
| IL-12β | Dendritic cells | C17.8 | Bio X Cell | BE0051 | AF488 |
| TNIK | TNIK in cells | NA | Novus Biologicals | NBP1-82994 | αRb (AF555) |
| GFP | Tumor cells | NA | Invitrogen | A11122 | AF647 |
| Rabbit IgG | Control | 6B9G9 | Biolegend | 410404 | AF555 |

## Table S2. Antibodies used for flow cytometry

| **Marker** | **Host** | **Vendor** | **Catalog #** | **Fluorophore** |
| --- | --- | --- | --- | --- |
| CD45 | Rat | Biolegend | 103114 | PC-Cy7 |
| CD11b | Rat | Biolegend | 101212 | APC |
| TCRb | Hamster | Biolegend | 109208 | PE |
| CD4 | Rat | BD | 563232 | BV650 |
| CD8 | Rat | Biolegend | 100714 | APC-Cy7 |
| IFNg | Rat | Biolegend | 505824 | AF700 |
| PD1 | Rat | Biolegend | 135217 | BV421 |
| CD19 | Rat | Biolegend | 152406 | PerCP/Cy5.5 |
| FoxP3 | Rat | BD Pharmigen | 562466 | PE-TexasRed |
| TNIK | Rabbit | Novus Biologicals | NBP1-82994 | unconjugated |
| CD25 | Rat | Biolegend | 102024 | AF700 |
| CD69 | Hamster | Biolegend | 104507 | PE |
| CD44 | Rat | Invitrogen | 67-0441-82 | BV711 |
| CD62L | Rat | Biolegend | 104403 | Biotin-Strep-BX421 |
| F4/80 | Rat | Biolegend | 123146 | PE/Dazzle594 |
| CD11c | Hamster | Biolegend | 117324 | APC-Cy7 |
| Ly6G | Rat | Biolegend | 127643 | BV711 |
| Ly6C | Rat | Biolegend | 128024 | AF700 |
| MHCII (I-A/I-E) | Rat | Biolegend | 107620 | BV421 |
| NK1.1 | Mouse | Biolegend | 108727 | PerCP |
| HMGB1 | Mouse | BioLegend | 651406 | PE-TexasRed |
| CALR | Mouse | Novus | NBP1-47518AF700 | AF700 |
| Rabbit IgG | Donkey | Biolegend | 406419 | BV510 |
